# A phased genome of the highly heterozygous ‘Texas’ almond uncovers patterns of allele-specific expression linked to heterozygous structural variants

**DOI:** 10.1093/hr/uhae106

**Published:** 2024-04-09

**Authors:** Raúl Castanera, Carlos de Tomás, Valentino Ruggieri, Carlos Vicient, Iban Eduardo, Maria José Aranzana, Pere Arús, Josep M Casacuberta

**Affiliations:** Centre for Research in Agricultural Genomics, CRAG (CSIC-IRTA-UAB-UB), Campus UAB, 08193, Cerdanyola del Vallès, Barcelona, Spain; Centre for Research in Agricultural Genomics, CRAG (CSIC-IRTA-UAB-UB), Campus UAB, 08193, Cerdanyola del Vallès, Barcelona, Spain; Biomeets Consulting, Carrer d’Àlaba 61, 08005, Barcelona, Spain; Centre for Research in Agricultural Genomics, CRAG (CSIC-IRTA-UAB-UB), Campus UAB, 08193, Cerdanyola del Vallès, Barcelona, Spain; Centre for Research in Agricultural Genomics, CRAG (CSIC-IRTA-UAB-UB), Campus UAB, 08193, Cerdanyola del Vallès, Barcelona, Spain; IRTA (Institut de Recerca i Tecnologia Agroalimentàries), 08140, Caldes de Montbui, Barcelona, Spain; Centre for Research in Agricultural Genomics, CRAG (CSIC-IRTA-UAB-UB), Campus UAB, 08193, Cerdanyola del Vallès, Barcelona, Spain; IRTA (Institut de Recerca i Tecnologia Agroalimentàries), 08140, Caldes de Montbui, Barcelona, Spain; Centre for Research in Agricultural Genomics, CRAG (CSIC-IRTA-UAB-UB), Campus UAB, 08193, Cerdanyola del Vallès, Barcelona, Spain; IRTA (Institut de Recerca i Tecnologia Agroalimentàries), 08140, Caldes de Montbui, Barcelona, Spain; Centre for Research in Agricultural Genomics, CRAG (CSIC-IRTA-UAB-UB), Campus UAB, 08193, Cerdanyola del Vallès, Barcelona, Spain

## Abstract

The vast majority of traditional almond varieties are self-incompatible, and the level of variability of the species is very high, resulting in a high-heterozygosity genome. Therefore, information on the different haplotypes is particularly relevant to understand the genetic basis of trait variability in this species. However, although reference genomes for several almond varieties exist, none of them is phased and has genome information at the haplotype level. Here, we present a phased assembly of genome of the almond cv. Texas. This new assembly has 13% more assembled sequence than the previous version of the Texas genome and has an increased contiguity, in particular in repetitive regions such as the centromeres. Our analysis shows that the ‘Texas’ genome has a high degree of heterozygosity, both at SNPs, short indels, and structural variants level. Many of the SVs are the result of heterozygous transposable element insertions, and in many cases, they also contain genic sequences. In addition to the direct consequences of this genic variability on the presence/absence of genes, our results show that variants located close to genes are often associated with allele-specific gene expression, which highlights the importance of heterozygous SVs in almond.

## Introduction

Almond [*Prunus dulcis* (Miller) D.A. Webb, syn. *P. amygdalus* (L) Batsch] is the most important tree nut crop in terms of commercial production and in the last years its production has duplicated, arriving in 2021 to 1.76 kernel million tons worldwide. In addition to its economic importance, almond shows a high nutritional value and a high adaptability to different environments and irrigation regimes. Almond belongs to the *Rosaceae* family and the *Prunus* genus, together with other important species as peach (*P. persica*), apricot (*P. armeniaca*), sweet cherry (*P. avium*), Japanese plum (*P. salicina*), European plum (*P. domestica*), and sour cherry (*P. cerasus*).

Unlike for the other species of the genus, almond was domesticated for its seed or kernel and not for its fleshy mesocarp. Modern almond breeding started in the 1920s. The first breeding programs were based on classical breeding, making controlled crosses to develop new almond varieties with superior performance and being self-compatible and with a late blooming date. Nowadays, many traditional cultivars have been or are being substituted by these new commercial gene pool with a lower genetic diversity [1].

Almond marker–assisted breeding is in its infancy and very few traits as self-compatibility, sweet kernel, and late blooming are being selected with molecular markers [1]. To speed up this process, in the last years, three reference genomes of three different cultivars, cv. Lauranne [2], cv. Texas [3], and cv. Nonpareil [4], and a 60K almond SNP array [5] have been published. These whole genome sequences, together with others from other *Prunus* species including peach [6], sweet cherry (*P. avium* L.) [7], mume (*P. mume* L.) [8], apricot (*P. armeniaca*) [9], Japanese plum [10], and *P. yedoensis* [11] have been made available in the Genome Rosaceae Database [12].

While self-compatibility is a common feature of modern almond varieties [1], most traditional almond varieties are self-incompatible and, as a consequence, the level of variability of almond is very high. As none of the available reference genome sequences of *Prunus* species, including almond, is phased, they are in all cases a collapsed representation of the genome of the sequenced genotype. However, interallelic interactions are crucial to understand phenotypic variation and heterosis [13] and therefore, having information of the different haplotypes and their interactions is essential in order to understand the genetic basis of trait variability. Here, we present a phased assembly of genome of the almond cv. Texas. In addition of the haplotype information, this new assembly, which we have called Texas v.3.0, has 13% more assembled sequence than the previous version of the Texas genome (Texas v.2.0) [3]. Our analysis shows that the Texas genome has a high degree of heterozygosity, both as SNP, short indel, and structural variant (SV) level. These SVs are frequently related to heterozygous TE insertions and in some cases also contain genic sequences. We show here that the variants predicted of high impact tend to be associated with allele-specific expression (ASE), which highlights their importance on gene regulation.

## Results

### An improved and phased assembly of Almond cv. Texas genome

We used a combination of 260X coverage of PacBio long reads and 172X coverage Hi-C Illumina short reads for genome assembly. Our pipeline included Falcon-unzip to assemble contigs and phased associated contigs (haplotigs), followed by polishing, duplicate purging, scaffolding, and phasing (Supplementary Fig. S1). We assembled a total of 250 Mb in 362 primary contigs and 128 Mb in 1345 haplotigs, and Hi-C data were used to fix phase switches between haplotigs within primary contigs. Chromosome-scale assembly was achieved by mapping the resulting 80 scaffolds and 99 additional contigs to linkage groups using the genetic map of *Texas* × *Earlygold* [14] and Texas v.2.0 assembly. As a result, we obtained a phased genome assembly (Texas v.3.0) that spanned 254.02 Mb for phase-0 (hereafter referred as P0) and 252.65 Mb for phase-1 (hereafter referred as P1), containing 97.3% of the total contig sequence anchored to eight pseudomolecules. In comparison to the previously available Texas v.2.0 reference genome [3], which is a collapsed representation of the two haplotypes, the Texas v.3.0 assembly contains up to 13.2% more contig sequence (30 Mb more for P0 and 29.93 Mb for P1). This increase in assembled sequence is homogeneously distributed among the eight chromosomes and is accompanied by a concomitant reduction of unplaced contig sequence (Supplementary Fig. S2). The sequence contiguity is strongly improved, with an average of 11.5× higher contig N50 with respect to Texas v.2.0 (Table 1), and this improvement correlates with a strong increase of the LTR Assembly Index (LAI) score, a common indicator of assembly quality (Table 1), with a figure that corresponds to the category of ‘gold quality genome’ as proposed by the developers [15]. The results of BUSCO (Benchmarking Universal Single-Copy Orthologs) [16], evidenced an increased completeness at the gene level in comparison to Texas v.2.0, with 96.9 and 97.7% of BUSCO complete genes in P0 and P1, respectively (95.4% in Texas v.2.0), and <2% of BUSCO missing genes (Table 1).

**Table 1:** Genome assembly and annotation statistics

**Feature**	**Texas v.3.0 Phase 0**	**Texas v.3.0 Phase 1**	**Texas v.2.0**
Assembly length (Mb)	254.02	252.65	227.59
Pseudomolecule N50 (Mb)	30.53	30.47	24.8
Contig	362	362	4395
Contig L50	62	61	511
Contig N50 (Mb)	1.21	1.19	0.104
Max. Contig length (Mb)	7.01	7.01	1.31
Percent anchored to pseudomolecules	98	98	91.47
Gap (%)	0.01	0.01	1.72
LAI index	20.58	20.92	8.15
BUSCO complete genes (%)	96.9	97.7	95.4
BUSCO fragmented genes (%)	1.4	0.9	1.0
BUSCO missing genes (%)	1.7	1.4	3.6
Number of protein-coding genes	28 625	29 616	27 969
Genes with Pfam domain^*^	22 892 (79%)	23 413 (79%)	21 582 (77%)
Gene density (genes/Mb)	113	117	123
Mean CDS length	1153	1122	1244
Mean exons per transcript	5.3	5.3	5.4

^*^e-value <0.05 | FDR < 5%.

**Figure 1:**
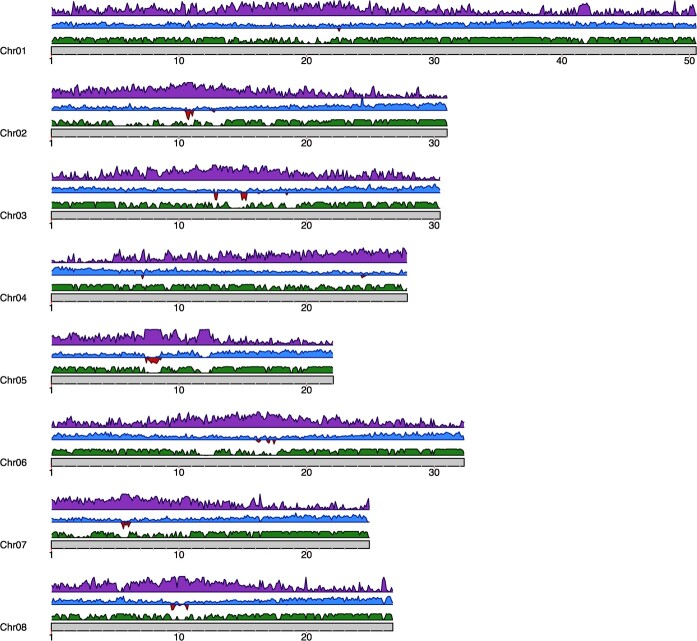
Density of genomic features of *P. dulcis* Texas v.3.0 chromosome-level assembly. Green tracks (lower tracks) represent the genome alignment of Texas v.2.0 against Texas v.3.0. Centromeric repeats are shown in red (middle tracks, facing downwards), genes in blue (middle tracks, facing upwards), and transposable elements in purple (upper traks). Density was calculated in windows of 100 Kb of sequence.

As expected, the new assembly shows high overall collinearity with that of Texas v.2.0 (Supplementary Fig. S3). However, we found some structural variations when comparing P1 and P0 against Texas v.2.0, being 95% insertions and deletions with a mean size of 1015 bp (Supplementary Table S1). A search for the 166-bp centromeric repeat previously described for *Prunus* species (including almond) [17] showed that the number of copies of this repeat is 8.9-fold higher in Texas v.3.0 with respect to Texas v.2.0, indicating a much better assembly of centromeric regions. The 166-bp centromeric repeat sequence localizes in sharp single peaks in five out of the eight chromosomes, which potentially correspond to the centromeres (Fig. 1). This result and the fact that the regions surrounding the 166-bp centromeric repeat peaks in Texas v.3.0 contain a high number of LTR-retrotransposons (LTR-RT) that were not annotated in Texas v.2.0 (see below) suggest that the structure of the centromeres is much better resolved in Texas v.3.0 as compared with Texas v.2.0.

In general, highly repetitive regions, containing tandemly repeated genes and transposable elements (TEs) are more difficult to assemble using short-read-based approaches, as it was the case of the Texas v.2.0 assembly, and are better assembled in this new Texas v3.0 assembly. As an example, we show the locus *Vr3* containing the powdery mildew resistance gene in almond [18]. As it can be seen in Supplementary Fig. S4, this locus was heavily scrambled in the Texas v.2.0 assembly whereas it appears as highly syntenic with peach in the Texas v.3.0 assembly, suggesting that the structure of this locus is now better resolved. The gene annotation (see below) of this region resulted in 10 newly annotated genes in Texas v3.0 assembly (Supplementary Data S1). This new assembly and genome annotation should help determining the gene underlying the *Vr3* resistance to powdery mildew.

### Gene annotation

We generated transcriptomic data from almond cv Texas flower and fruit to complement the previously reported leaf data [19], and we used them to assist gene annotation together with available transcriptomic data from different tissues and conditions from other almond cultivars, a public *P. dulcis* and *P. persica* protein collection (Supplementary Table S2), as well as *ab initio* gene prediction (Supplementary Fig. S5). We annotated 29 616 protein-coding genes and 534 tRNAs on Phase-1, of which 96.7% could be successfully lifted to Phase-0. We used Liftoff to map the annotation of Texas v.3.0 to Texas v.2.0, and found that Texas v.3.0 contains 2518 additional genes as compared with the gene annotation of Texas v.2.0, 79.7% of them harboring a PFAM conserved domain. The most abundant functions of the proteins encoded by these genes are ubiquitin-like proteases (257), FAR1-related proteins (61), disease resistance proteins (59), and putative transcription factors (13). When mapping the Texas v.2.0 annotation to the new assembly, 26 116 genes (96.6%) were successfully lifted to at least one of the two Texas v.3.0 phases, with an average gene coverage of 99.1% and an average identity of 98.7%. Nevertheless, we identified 926 genes that failed to be lifted (77.3% of them carrying PFAM domain). The most abundant functions among those genes are protein kinases (76) and leucine-rich repeat domain containing proteins (67).

### Texas v.3.0 identifies recent TE insertions missing in the previous assembly version

**Figure 2:**
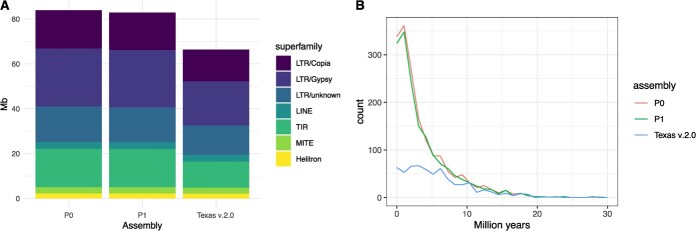
**A** TE content in P0, P1, and Texas v.2.0 assemblies (Mb of sequence). **B** Estimated insertion age of intact LTR-retrotransposons in P0, P1, Texas v.2.0, and peach reference genome.

We annotated TEs using a combination of structural and homology-based approaches (see Methods). This general TE annotation was complemented with a set of complete elements containing both structural and coding TE domains, to produce the final TE annotation. As expected, and as previously described for Texas v.2.0 [3], TEs and genes show an opposite overall distribution along chromosomes (Fig. 1), with TEs concentrating in low gene-density regions such as the regions surrounding the putative centromeres (Fig. 1). TE sequences account for 32.9% of the assembled sequence in both P0 and P1 of Texas v3.0, which represents an additional 17 Mb of annotated TE sequences (per phase) as compared with Texas v.2.0 (Fig. 2A). Each of the Texas v.3.0 phases contain about twice the number of complete TE elements as compared with Texas v.2.0 (Table 2), which reflects the higher contiguity of this version of the Texas genome.

**Table 2:** TE content in *P. dulcis* Texas

	**Percentage of genome size (%)**			**Number of complete elements** ^**a**^		
**Order/Superfamily**	**P0**	**P1**	**Texas v.2.0**	**P0**	**P1**	**Texas v.2.0**
TIR	6.7	6.7	5.0	355	348	240
MITE	1.1	1.1	1.2	620	624	511
Helitron	0.9	0.9	0.9	9	9	10
LTR/Gypsy	10.2	10.1	8.6	519	479	126
LTR/Copia	6.7	6.6	6.2	790	743	340
LTR/Unknown	6.2	6.2	5.7	468	464	236
LINE	1.2	1.2	1.3	*NA*	*NA*	*NA*
Total	33.0	32.8	29.2	2761	2667	1463

a Containing structurally intact features (see Methods).

This increase is particularly important for complete Gypsy LTR-RTs as their number in Texas v3.0 is 3-fold that of Texas v2.0. This could be due to the already-mentioned improved assembly of the regions putatively containing the centromeres, as Gypsy LTR-RTs tend to concentrate in these regions of plant genomes [20]. In order to investigate this, we performed whole-genome alignments of Texas v.2.0 to Texas v.3.0 and looked for Gypsy LTR-RTs in Texas v.3.0-specific regions. We found that 50.6% of the complete elements are present in regions that are absent from the Texas v.2.0 assembly. In addition, when we looked at the distribution of all Gypsy LTR-RTs (including incomplete elements) absent in Texas v2.0, we found a strong enrichment in the potential centromeric regions (Supplementary Fig. S6), confirming that the increase in Gypsy LTR-RTs in Texas v.3.0 as compared with Texas v.2.0 is mainly due to the better assembly of the centromeric and pericentromeric regions. In addition, the new assembly contains many more complete Copia LTR-RT elements, as well as more complete TIR transposons (Table 2), which are more evenly distributed throughout the chromosomes (Supplementary Fig. S7). An analysis of the age of LTR-RTs inferred from the intra-element LTR comparison showed that an important fraction of the LTR-RTs newly annotated in this assembly are young LTR-RT insertions (<5 My) (Fig. 2B).

Within the TIR order, the most important differences between the two assemblies were found in the EnSmp/CACTA and MuDR superfamilies. In particular, the new assembly contained a 2.5-fold increase in EnSmp/CACTA sequence over Texas v.2.0 (5.4 Mb vs 2.2 Mb). An analysis of the divergence of every TIR copy versus its respective TE consensus sequence, which can be used as an indication of the element’s age, revealed that a major fraction of the EnSmp/CACTA elements newly annotated in this assembly are young elements, similarly to what we found for LTR-RTs (Supplementary Fig. S8).

### Almond haplotypes harbor genetic variation with potentially high functional impact

**Figure 3:**
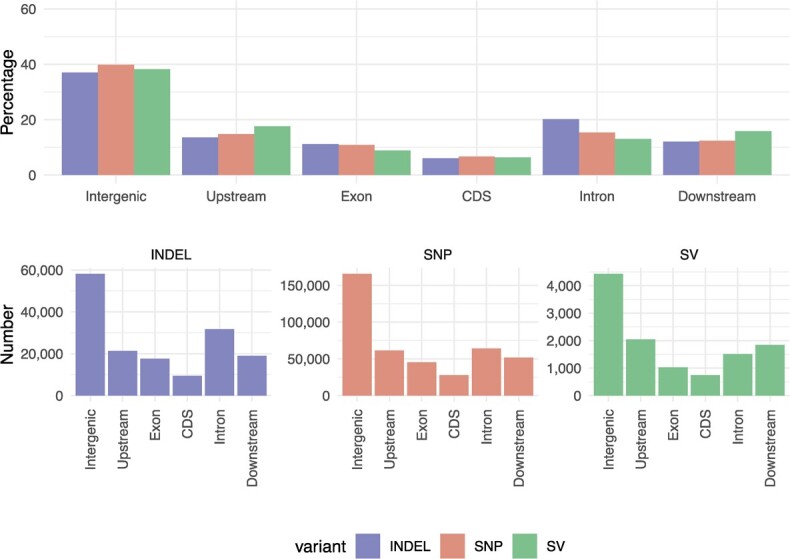
Percentage and number of indels, SNPs, and SVs on the different genomic features. Upstream/Downstream regions = 1 Kb.

We compared the two phases of Texas v.3.0 by aligning P0 to P1 sequence, and found 365 176 SNPs, 138 897 INDELs (<40 bp), and 8294 structural variants (SVs, length ≥40 bp). Considering the 128 Mb spanned by the haplotigs, this represents one SNP every 350 bp, one INDEL every 921 bp, and one SV every 15 432 bp of phased sequence. We found that SVs were proportionally more abundant than other variants in upstream and downstream gene regions (1 Kb), whereas indels were more abundant in introns and SNPs in CDS and intergenic regions (Fig. 3). SNP and INDELs were further classified according to their predicted coding impact using snpEff [21](low, moderate, or high). INDELs showed proportionally a higher coding impact than SNPs, (4.72% vs 0.11% of their effects classified as high impact, respectively). In total, we identified 4348 genes carrying variants with predicted high coding impact, the vast majority (90%) of them being INDELs. Most of those genes (4035) contain frameshift variations, and 501 contain gain or loss of a stop codon. In addition to SNPs and INDELs, we found 2046 SVs overlapping potential gene regulatory regions (1 Kb upstream of genes) and 714 affecting coding sequences. Among those, we found 357 genes completely included in deletions in Phase-0. A subset of these presence–absence variation (PAV) genes have unknown/uncharacterized functions (28%). Among the rest, the most abundant functions were ULPs (ubiquitin-like proteases) and Ankyrin repeat family proteins, although we found no significantly enriched gene ontology (GO) terms.

### TEs are at the origin of a major fraction of the heterozygous structural variation

Among the 8294 SVs detected, the vast majority (93.6%) were insertions and deletions, although we also detected 14 interspersed duplications, 100 tandem duplications, and 5 small inversions (ranging from 5 to 80 Kb). The size of the insertion/deletion SV ranged from 40 bp (the arbitrary lower threshold to be considered an SV) to 66 230 bp, with three peaks around 70 bp, 600 bp, and 6 Kb (Supplementary Fig. S9). An important fraction of the insertions/deletions (32%) overlap almost perfectly with a TE annotation (intersect >80% of TE length and >50% of SV length), in particular for the large insertions/deletions, suggesting that they correspond to heterozygous TE insertions (1314 specific of P0 and 1258 specific to P1). A visual inspection of a subset of these SV potentially related to LTR-RTs (i.e. all the SV appearing as deletions in P1 in chromosome 5 and overlapping with a Gypsy or Copia LTR-RT complete copy in phase-0, *n* = 18) showed that in all cases, the insertion corresponds to an LTR-RT element plus 5 nt (Supplementary Fig. S10). As the target site duplication (TSD) typically generated by LTR-RTs is of 5 nt, the analyzed SVs perfectly correspond to a typical LTR-RT insertion. We therefore conclude that most, if not all, of the 32% of SVs with high overlap with TEs are the consequence of TE insertions/deletions. Moreover, we identified an additional 29% of SVs that overlap with TEs only partially. These cases are not likely the result of transposition, but may be the result of TE internal deletions or rearrangements. In any case, this suggests that a major fraction of the heterozygous structural variation is TE-related.

We detected heterozygous TE insertions from all the different TE orders, with LTR-RTs being the most abundant (66% of the total) (Supplementary Table S3). Given that the Texas v.2.0 is a collapsed representation of the two haplotypes, we hypothesized that heterozygous TE insertions may be underrepresented in this unphased assembly, thus explaining the difference in the number of TEs between Texas v.3.0 and Texas v.2.0. Indeed, we detected 3148 TEs insertions that are missing in Texas v.2.0 assembly (Texas v3.0 TEs loci that are empty in Texas v.2.0, Supplementary Fig. S11) and found that 94.1% of them are heterozygous TE insertions. This suggests that for regions harboring heterozygous TE insertions, the empty haplotype was more frequently included in the Texas v.2.0 assembly.

An analysis of the insertion time of LTR-RTs showed that the heterozygous insertions were in general more recent than the homozygous ones (mean heterozygous = 2.6 Mya, homozygous = 6.5 Mya, Wilcoxon *P* < 0.05, Supplementary Fig. S12A) and therefore have had less time to become fixed or being eliminated since their insertion. On the other hand, an analysis of presence/absence of in a collection of 40 almond accessions using publicly available short-read data (Supplementary Table S4) shows that the heterozygous insertions are present at a much lower population frequency than the homozygous ones (mean heterozygous = 0.32, mean homozygous = 0.90, Supplementary Fig. S12B). However, a small fraction of the heterozygous insertions are present at high frequencies in the population and are relatively old and have been maintained unfixed in the population.

### Relationship between TE insertions and gene expression

**Figure 4:**
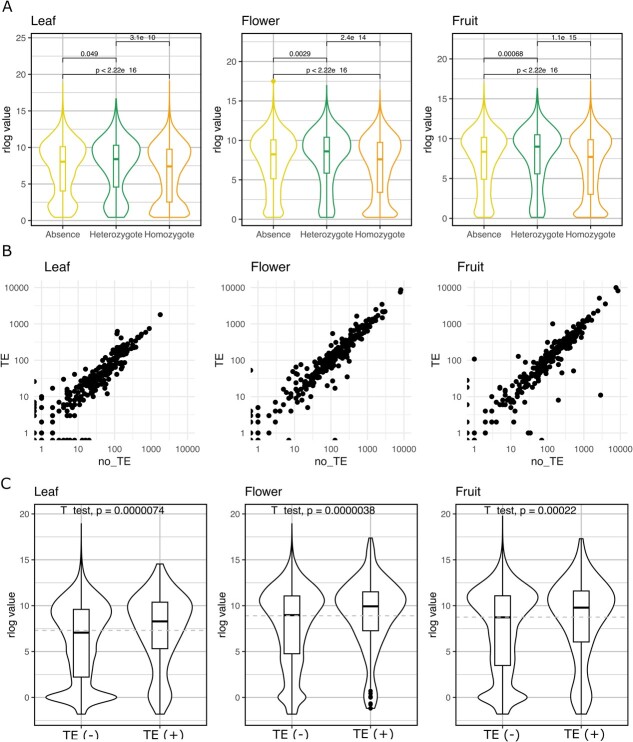
**A** Relationship between gene expression levels (log scale) and the presence of homozygous and heterozygous TE insertions at <1-Kb upstream gene TSS. **B** Allelic expression of 284 genes carrying a heterozygous TE insertion at <1 Kb in the upstream region (counts of TE and non-TE alleles). **C** Expression levels of peach genes without heterozygous TE insertion [TE(−), *n* = 22 134] or with heterozygous TE insertion [TE(+), *n* = 200] in the promoter of its almond ortholog. Expression in the *Y*-axes of all panels is presented as DEseq2 regularized log values.

The analysis of the distance between TE insertions and genes shows that heterozygous insertions are in general closer to genes as compared with the homozygous insertions (median of 1.7 vs 3.0 Kb, respectively, Supplementary Fig. S13). As in general the heterozygous insertions are younger, this may suggest an impact on gene coding or expression capacity of these TE insertions that are purged by selection with time. To test the potential impact of TE insertions on gene expression, we used the RNASeq data from almond (cv. Texas) from immature fruits, flowers, and leaves (see Methods for details). We compared the expression of genes that do not contain a TE insertion in the proximal upstream region (1 Kb) with that of genes carrying a homozygous or heterozygous TE in this region. We observed the same pattern in the three RNASeq datasets obtained from different organs. In all cases, genes with homozygous TE insertions had lower expression than genes without TEs (*P* < 0.05), which suggests that TE insertions in the upstream regions of genes have, in general, a negative effect on gene expression. Interestingly, this trend is reversed for the genes harboring a heterozygous insertion in the proximal upstream region, which have a higher expression level (*P* < 0.05) than those without a TE insertion (Fig. 4A). This could suggest an opposite impact of homozygous and heterozygous TE insertions, with heterozygous insertions activating gene expression. In order to test this hypothesis, we analyzed the allele-specific expression of the 284 genes containing a heterozygous TE insertion in the upstream proximal region (1 Kb). Our data show that in general, the allele without the TE insertion tends to be expressed at similar level than the one containing the insertion (Fig. 4B), although in some specific cases, the presence of the heterozygous TE is linked with increased or reduced expression levels. The higher expression of genes with heterozygous TE insertions could also be due to a preference of insertion of TEs into genes that are highly expressed. This association of TE insertions with highly expressed genes could then be lost with time due to purifying selection, or due to the overall negative effect of the fixed TE insertions on gene expression. To test this hypothesis, we produced transcriptomic data from different peach organs (divergence time from common ancestor = 5.88 million years) and asked for the expression of peach genes devoid of TE insertions in the promoter but whose almond ortholog has a heterozygous TE insertion in the promoter region (indicator of recent insertion in almond). Our results clearly show that peach genes whose almond orthologs have a heterozygous TE insertion are expressed at a higher level than the mean expression of peach genes (Fig. 4C).

### Allele-specific expression patterns in almond are associated with nearby SVs

**Figure 5:**
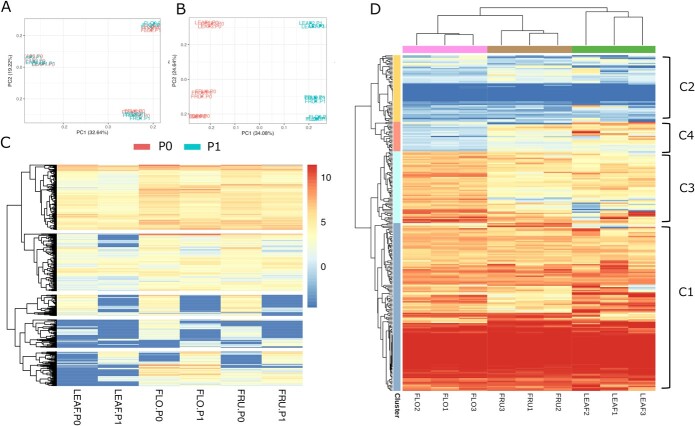
**A** PCA of leaf, flower, and fruit samples based on the allele expression levels of 6182 genes with detectable allele-specific expression. **B** PCA of leaf, flower, and fruit samples based on the allelic expression levels of the 579 genes with differential expression between alleles in at least one organ. **C** Heatmap representing the allele-specific expression (RNAseq counts in logarithmic scale) of 579 genes with allele-specific expression. Each row represents a gene. **D** Heatmap representing the allele-specific expression profiles of 250 genes with at least 10 mapped reads in every replicate. Colors indicate the percentage of mapped reads from each allele (red = 100% P0, blue = 100% P1) over the total. Co-expression clusters are marked by ‘C’ (C1 to C4).

To analyze the genome-wide patterns of allele-specific expression (ASE), we searched for SNPs present in gene coding regions without surrounding INDELS (at <50 bp) that could allow us to differentiate the expression of the two alleles. We found 24 051 SNPs fulfilling this requirement in up to 6939 genes, for which 6182 showed detectable expression in at least one of the organs tested (leaves, flowers, and immature fruit). We found that 579 genes (9.3% of the expressed genes with informative SNPs) showed ASE (*P* < 0.05, FDR 5%) in at least one organ (82 in leaf, 493 in flower, and 271 in fruit). Only a small number of genes (68) showed ASE in the three organs, whereas 383 genes displayed ASE in a single organ. PCA performed with the independent expression of the two alleles of all detected genes (6182) clearly separated samples by organ and grouped together the haplotypes (Fig. 5A).

When we used for PCA with the 579 genes with ASE, PC1 separated samples by haplotype (P0 or P1) and PC2 by organ. The heatmap of allelic expression (Fig. 5C) revealed gene clusters expressed only in one of the two alleles, whereas in other clusters, both alleles are expressed at different levels. The heatmap also shows that the profile of leaf samples differs from that of flower and fruit, most likely due to their lower coverage. In order to minimize the possible bias introduced by differences of coverage on the RNASeq data of the three organs, we extracted the genes displaying ASE and more than 10 reads mapping to target SNPs in the three replicates of each organ (250 in total). We performed a hierarchical clustering and found four clusters of co-expressed alleles (Fig. 5D). Clusters 1 and 2 represent genes where one of the alleles is predominantly expressed in all organs, suggesting that they contain heterozygous mutations strongly affecting gene expression. On the contrary, clusters 3 and 4 contain genes that express different alleles in different organs. For example, some genes of cluster 3 specifically express the P0 allele in flowers, whereas the reverse is found for some genes of cluster 4. This shows that heterozygous mutations can allow for an extra layer of regulation of gene expression that could widen the genetic plasticity of this species. To analyze the possible influence of structural variation close to genes with their ASE, we looked for the presence of SVs close to these genes. We found that 34.4% of the ASE genes contained a heterozygous SV at <1 Kb, in comparison to 28.0% found for non-ASE genes (*P* < 0.05, Fisher test). The proportion of ASE genes with structural variants in the two clusters where only one of the alleles was expressed in the three organs was 38.8% (Cluster 1) and 40.4% (Cluster 2). Nevertheless, the highest enrichment of SVs was found on Cluster 4, which contains genes showing ASE in an organ-specific manner (mainly in flower). In this cluster, 52.2% of the genes contained an SV in their surrounding regions.

## Discussion

Although in the last few years a number of almond genome assemblies have become available [2–4], the contiguity of their assemblies is variable, and all are a collapsed representation of the genome of this highly heterozygous species. We present here a more complete and phased assembly of the ‘Texas’ almond (Texas v3.0). The high contiguity of this assembly is particularly clear in genome regions difficult to assemble, such as the centromeres. These regions are much better resolved in Texas v.3.0 as compared with Texas v2.0, with an increased number of copies of the 166-bp centromeric repeat, which localizes in a single sharp peak in five out of the eight chromosomes, as well as the higher number of Gypsy LTR-RT annotated copies that colocalize with the 166-bp repeat. In addition of being a more complete assembly, Texas v.3.0, is also the first *Prunus* assembly that is phased. This is particularly relevant because almond is a highly heterozygous species. The analysis of the two haplotypes has allowed us to evaluate the heterozygosity level not only for SNPs but also for SVs and has revealed a high number of heterozygous TE insertions. Interestingly, a comparison of Texas v.2.0 and Texas v.3.0 TE annotations shows that an important number of the insertions annotated as heterozygous in Texas v.3.0 were not included in the previous version of the ‘Texas’ genome, suggesting a bias toward the empty allele in the collapsed representation of this genome. As the most recent TE insertions are overrepresented among the heterozygous TE insertions, the absence of these elements in the Texas v.2.0, suggested a more limited recent TE activity in almond as compared with peach [3], which is not supported by the analysis of the present Texas v.3.0 assembly.

The results presented here show that heterozygous TE insertions, as well as other structural variations between the two haplotypes, are linked to ASE, which affects 9.3% of the tested genes. In most cases, one allele is more expressed in all organs, but in few cases, there is alternative ASE in different organs, highlighting the wide range of phenotypic consequences of heterozygous SVs.

Although some TE insertions in the upstream regions of genes have been shown to result in gene activation (see, for example, Shi *et al*. [22]), TE insertions in gene promoters usually correlate with their repression. Our results are in line with a frequent negative impact of TE insertion close to genes, as we show that genes that contain homozygous TE insertions in the upstream regions are expressed at lower levels than those that do not contain a TE insertion. This is also the case of the heterozygous TE insertions, as in the subset of genes with ASE, the expression of the allele contain TE insertion is in general lower than that of the allele not containing the TE. However, an analysis of the overall expression of the genes that contain a heterozygous TE insertion shows that they are expressed at a higher level as compared with the genes not containing TEs in the vicinity. All these results suggest that TEs insert preferentially close to highly expressed genes and that their insertion modifies, often negatively, their expression. Many of these insertions will be purged by selection, but those that become fixed will be associated with a lower overall expression of the neighboring genes. Interestingly, the highly heterozygous nature of almond, and the fact that polymorphic TE insertions are maintained in the population for a long time [3], may allow almond to test the new pattern of expression that may have a positive impact under particular environmental conditions. This would be in line with recent results obtained in rice showing that TE insertions linked to changes in gene expression already present in wild populations can be selected in certain cultivated populations [23]. The presence of heterozygous TE insertions altering gene expression may be widespread in fruit trees, and it has been shown in apple [24] providing additional support to the important role of TEs as drivers of transcription variation impacting agronomic traits.

The phased genome sequence of ‘Texas’ almond is a qualitative improvement over the previous sequences of this species and other *Prunus*, strongly improving its completeness and contiguity and making possible the analysis of the variability carried by a single individual. The obtained sequence allowed us to ask questions on the transcriptomic consequences of structural variants, particularly those that are TE-based and located close to gene-coding sequences. Our results suggest that TEs tend to insert close to highly expressed genes and frequently have a negative impact on expression. Our results also show that these heterozygous TE insertions can be maintained for long periods of time. These results suggest that SVs may contribute to the generation and maintenance of phenotypically meaningful intrapopulation variability that may result in an enhanced adaptation capability to seasonal or long-term environmental changes.

## Methods

### Sampling, nucleic acids extraction, and sequencing

Leaves of *P. dulcis* cv Texas were collected from the IRTA Experimental Station of Lleida in Gimenells (Catalonia, Spain). High-molecular-weight genomic DNA was purified from the isolated nuclei as previously reported in Fiol *et al*. [25], using the Doyle CTAB (Cethyl Trimethyl Ammonium Bromide) method [26] and introducing an RNAse treatment before the chloroform centrifugation step. Total RNA was extracted using the Maxwell RSC Plant RNA Kit and the Maxwell RSC instrument (Promega Corporation, Madison, WI, USA). Complete DNA removal was obtained using the DNA-free DNA Removal Kit (Invitrogen™, Carlsbad, CA, USA).

### Genome assembly and phasing

PacBio reads were evaluated for quality and filtered using Filtlong (https://github.com/rrwick/Filtlong#full-usage) and Seqtk (https://github.com/lh3/seqtk). Illumina Hi-C reads were evaluated for quality using FastQC (http://www.bioinformatics.babraham.ac.uk/projects/fastqc/) and trimmed using Trimmomatic [27]. Falcon-unzip [28] was used to assemble PacBio reads into contigs and phased associated contigs (haplotigs), followed by polishing with Hapo-G [29] and duplicate purging with Purge_dups [30]. Hi-C reads were mapped to the assembly, and Phalcon-Unzip was used to fix phase switches between haplotigs within primary contigs. Scaffolding was performed to improve order and orientation of contigs using ALLHi-C pipeline [31]. Ordering and orientation of genomic scaffolds/contigs to reconstruct chromosomes was performed using ALLMAPS [32]. An additional integration of unassigned contigs and scaffolds (8% of the genome sequence) was performed by adding syntenic information coming from Texas v.2.0 assembly. A schematic overview of the whole pipeline is shown in Supplementary Fig. S1.

### Genome annotation

Gene annotation was performed in phase 1 using a custom pipeline based on MAKER, combining transcriptome-based, protein-based, and *ab initio*-based gene prediction (Supplementary Fig. S5). RNA-Seq datasets were retrieved from public and private collections spanning different tissues (Supplementary Table S2). Transcriptome assembly was performed by Trinity [33]following a genome-guided approach. Primary transcripts were selected with Evidentialgene pipeline (http://arthropods.eugenes.org/EvidentialGene/). To assign functional description, GO terms, and KEGG pathway information to the new gene models, sequences (transcripts/proteins) were functionally annotated with TRINOTATE [34].

### TE annotation

EDTA pipeline [35] was run independently on each Texas v.3.0 phase to obtain individual TE libraries and genome coordinates of LTR-retrotransposons. Redundancy between the two libraries was eliminated by running CD-HIT [36] at 80% identity cut-off. Unclassified consensuses and/or sequences with length <200 bp were filtered out, and the resulting library was complemented with LINE coding consensuses from the almond v2.0 TE annotation [3] to compensate EDTA low sensitivity on the detection of this TE order. A first round of RepeatMasker (https://www.repeatmasker.org/) was run using this preliminary TE library. LTR, LINE, and TIR consensuses that did not have a full-length RepeatMasker match (>80% of TE length) were removed. For MITEs and other non-coding consensuses elements, we only retained consensuses with three or more complete matches in the genome. Helitron consensuses without coding domains were filtered out too. Finally, the library was complemented with peach-specific coding consensuses (<80% identity based on CD-HIT clustering) obtained from Alioto *et al*. [3]. A second round of RepeatMasker was performed with the curated TE library and integrated with the EDTA structural annotation of intact LTR-retrotransposons. Specifically, LTR-retrotransposon RepeatMasker matches overlapping EDTA intact elements were removed from the final annotation.

### Identification of complete TE copies

We relied on elements retrieved from EDTA phase-1 as starting material to detect potential complete elements (*EDTA.intact.gff3 file). For LTR retrotransposons, we kept all intact elements due to the very low false-negative ratio (elements carrying LTR, TSD, and coding domains). For TIR and Helitrons, we extracted the sequence of all putative intact copies and re-classified them with Tesorter [37]. We kept elements where EDTA and Tesorter classification matched at the order level. MITE elements were extracted and clustered with CD-HIT at 80% identity. We kept only elements present in clusters of three or more copies.

### Identification of structural variants

We used Minimap2 [38] to align Texas F0 and F1 assemblies (parameters: -ax asm5), and svim-asm [39] was used to detect structural variants (default parameters). Heterozygous TEs were detected by performing a reciprocal intersection of TE annotations with the deletions found in P0 or P1 using bedtools. A TE was considered heterozygous if it spanned >50% of an overlapping deletion and the deletion covered at least 80% of the TE (bedtools parameters -F 0.5, -f 0.8). A TE was considered homozygous if it was completely covered by the genome alignment.

### Gene expression analyses

RNA-seq reads were mapped to almond or peach transcript models using Bowtie2 [40]. Alignment and transcript quantification were performed using RSEM (RNA-Seq by Expectation Maximization) algorithm with the Trinity [33] script ‘align_and_estimate_abundance.pl’. Statistical significance of transcript differences was tested with DESEQ2 [41].

### Allele-specific expression

Variants between P0 and P1 phases were detected using paftools.js *call* from Minimap2. We used Gatk4 [42], vcftools [43], and bedtools [44] to exclude indels and select only coding SNPs without INDELs in their proximity (at <50 bp). RNA-seq reads were mapped to P0 using Hisat2 [45]. Allele-specific counts were obtained using ASEReadCounter from GATK. Differential expression among alleles (ASE) was tested using DESEQ2 following the specific author recommendations for ASE (https://rstudio-pubs-static.s3.amazonaws.com/275642_e9d578fe1f7a404aad0553f52236c0a4.html).

## Supplementary Material

Web_Material_uhae106
