# Supplementary material for: A phased genome of the highly heterozygous ‘Texas’ almond uncovers patterns of allele-specific expression linked to heterozygous structural variants
Source: Hortic Res. 2024 Apr 9;11(6):uhae106. doi: 10.1093/hr/uhae106 (PMC11179849; doi:10.1093/hr/uhae106)
Supplement: Web_Material_uhae106 [file web_material_uhae106.zip › Supplementary figures and tables_revised.docx]

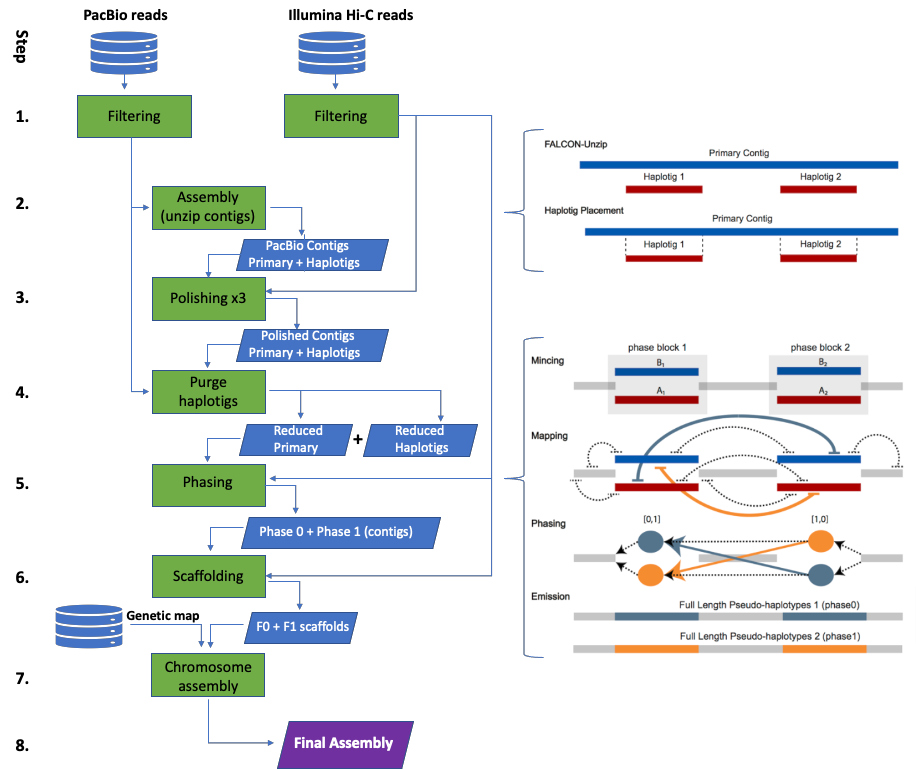


Supplementary Figure 1. Genome assembly pipeline

Supplementary Figure 2. Contig sequence per chromosome in Texas v3 phases (P0 and P1) and Texas v2 (Pdulcis26)


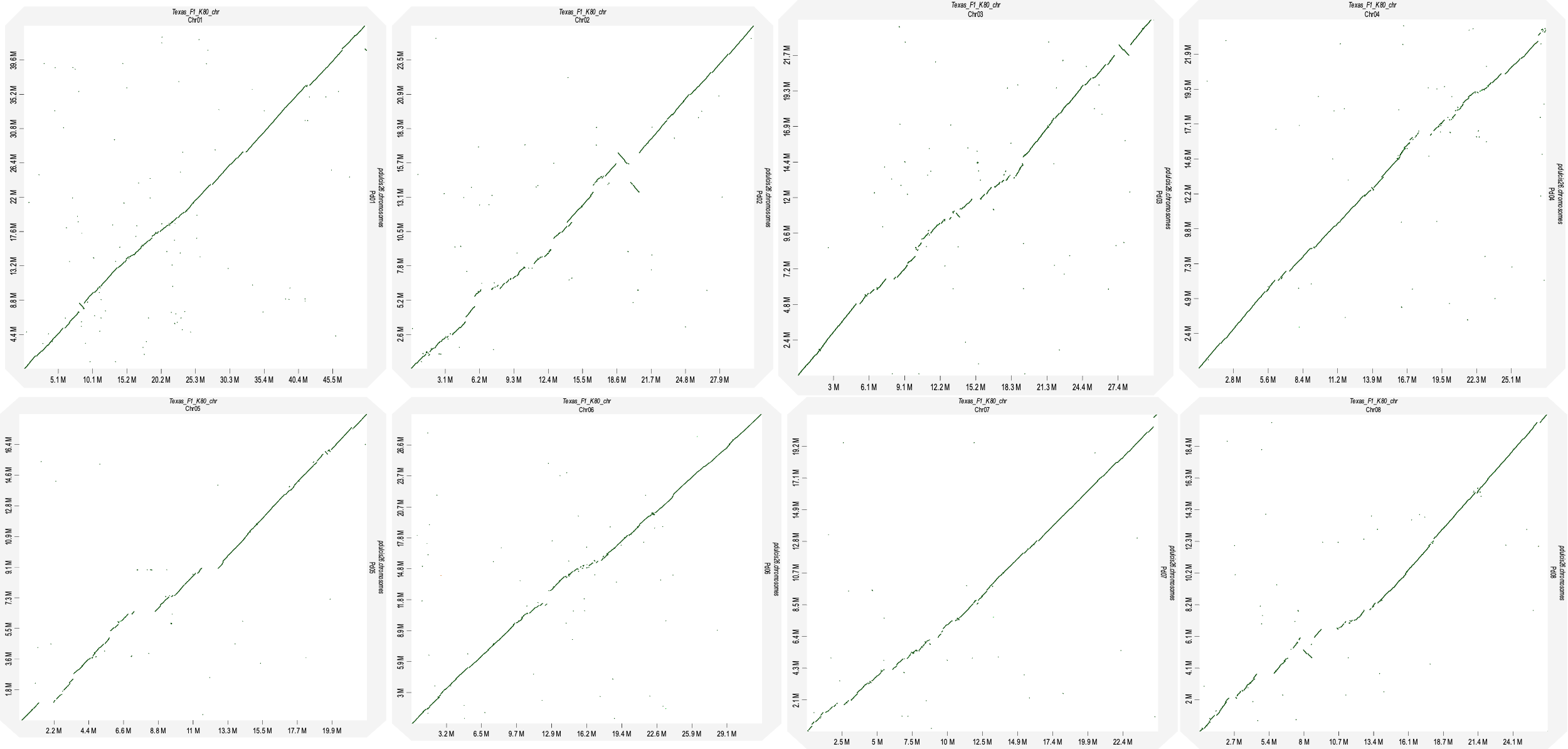


Supplementary Figure 3. Dot plot representation of the whole-genome alignment between Texas v.3.0 (Phase-1) and Texas v.2.0 pseudomolecules. Unplaced contigs are not shown.


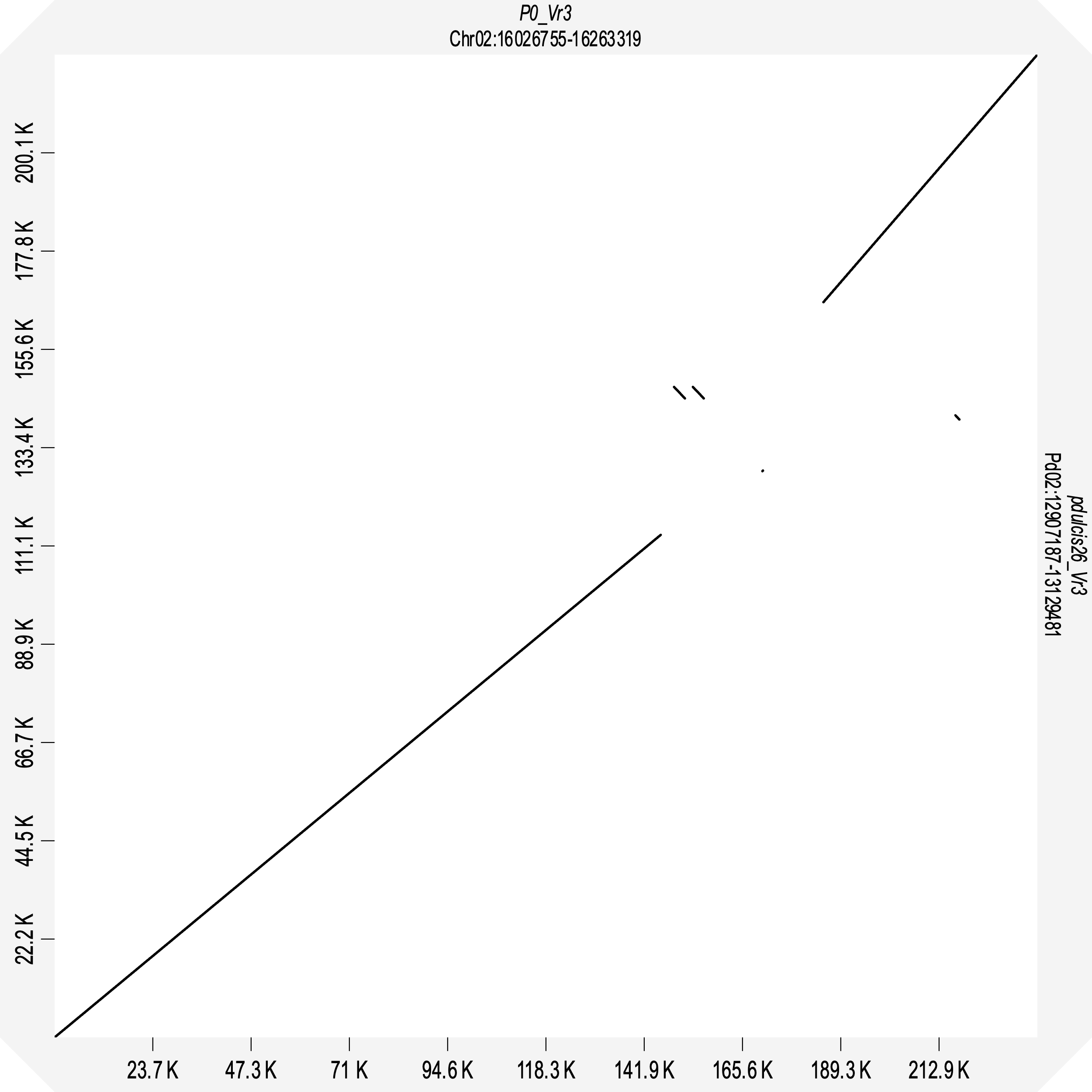

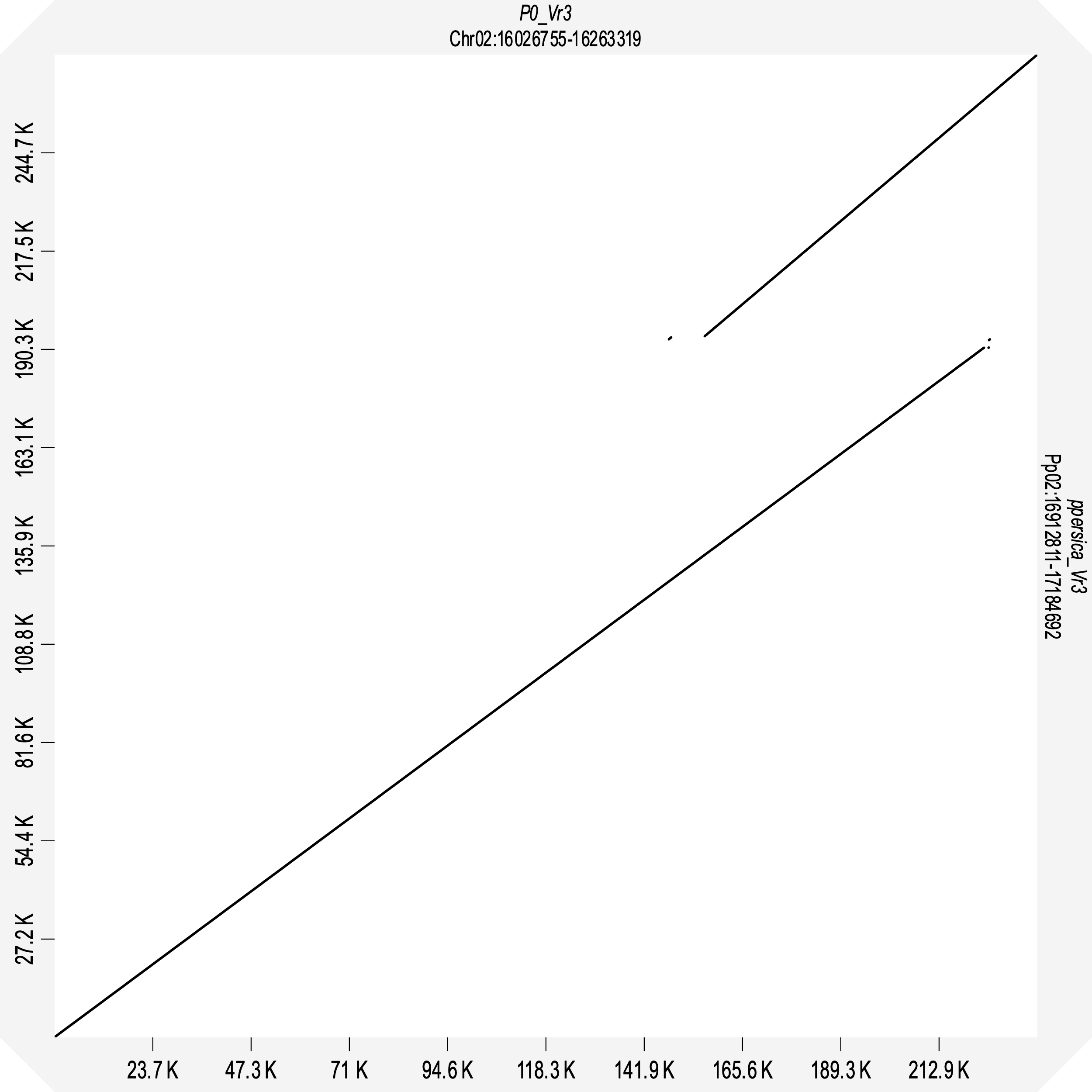

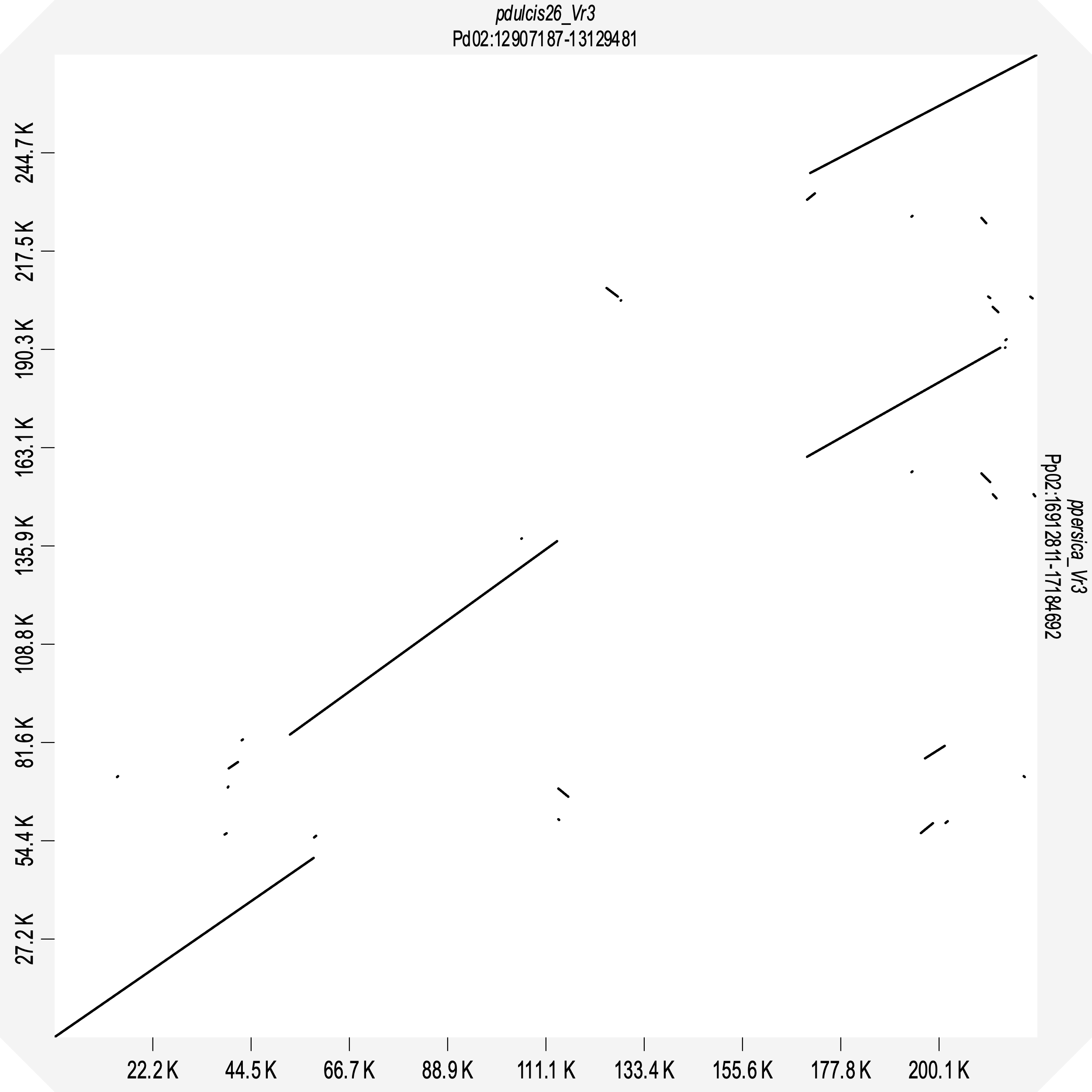


A

B

C

Supplementary Figure 4. Dotplot alignment of vr3 region present in chromosome 2: A) Texas v.3.0 (P0, horizontal) vs Texas v2.0 (vertical), B) Texas v.3.0 (P0, horizontal) vs Peach orthologous region (vertical), C) Texas v2.0 (horizontal) vs Peach genome (vertical). The new genome version is syntenic with that of peach, which carries a large duplication at the end of the region (B). By contrast, Texas v.2.0 version contains deletions, insertions, and unaligned regions (C).


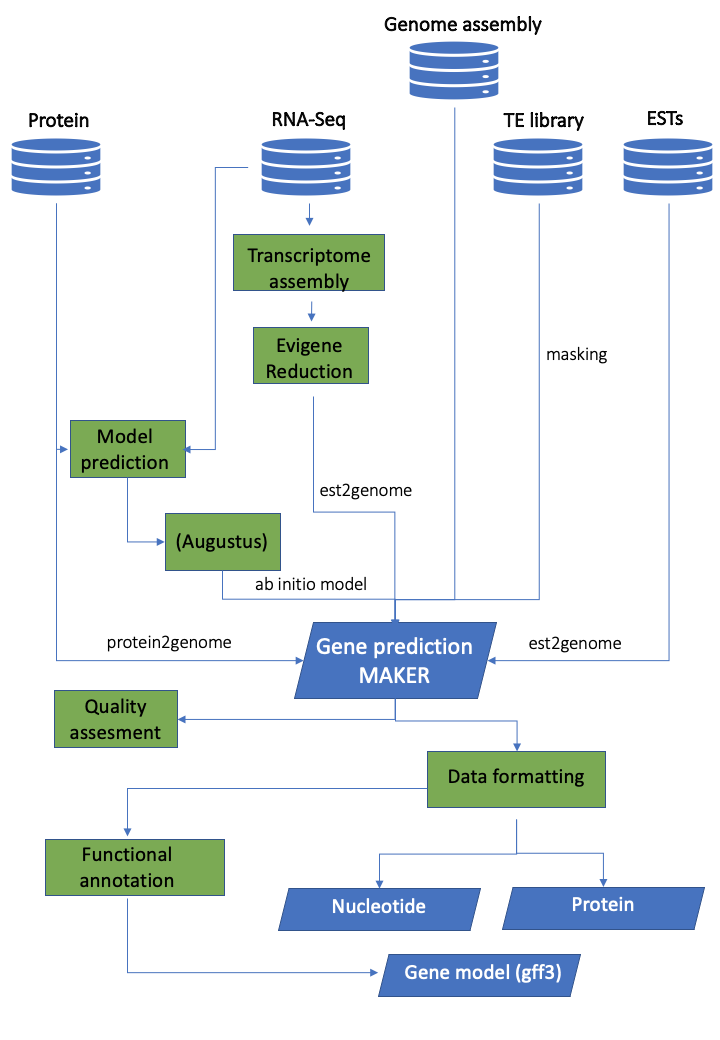


Supplementary Figure 5. Genome annotation pipeline.


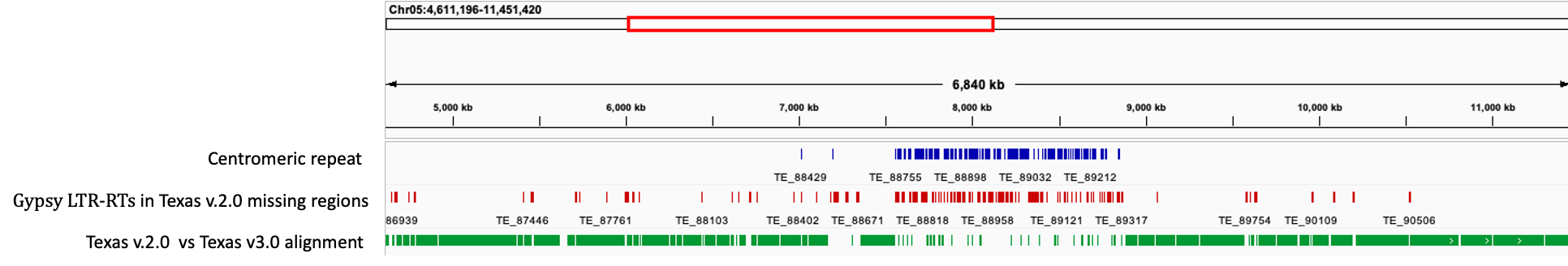


Supplementary Figure 6. Example of the enrichment of Texas v.3.0-specific Gypsy LTR-RTs in the potential centromere of Chr05. All these retrotransposons are absent in Texas v.2.0 assembly and fall in regions without genome alignment among the two assembly versions (in green).


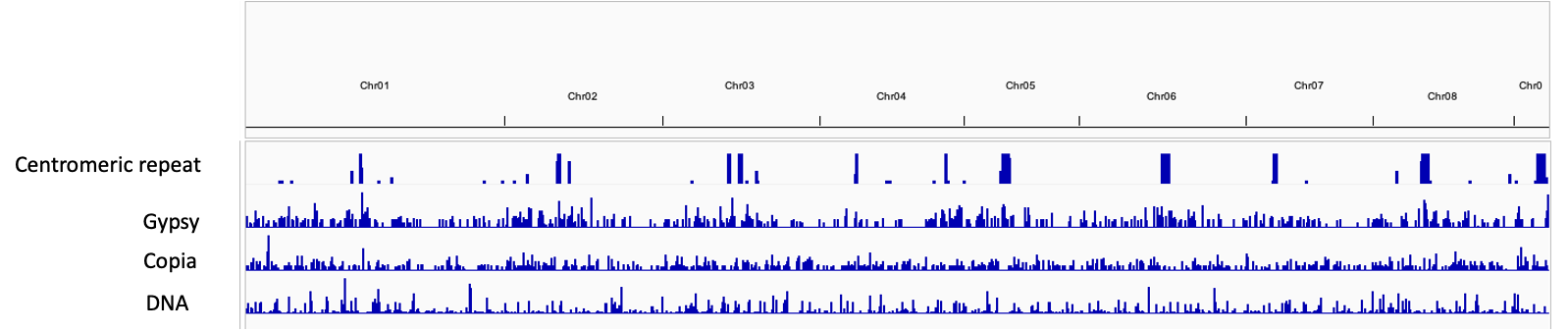


Supplementary Figure 7. Distribution of intact TE elements in the Texas v3.0 - P0 phase.


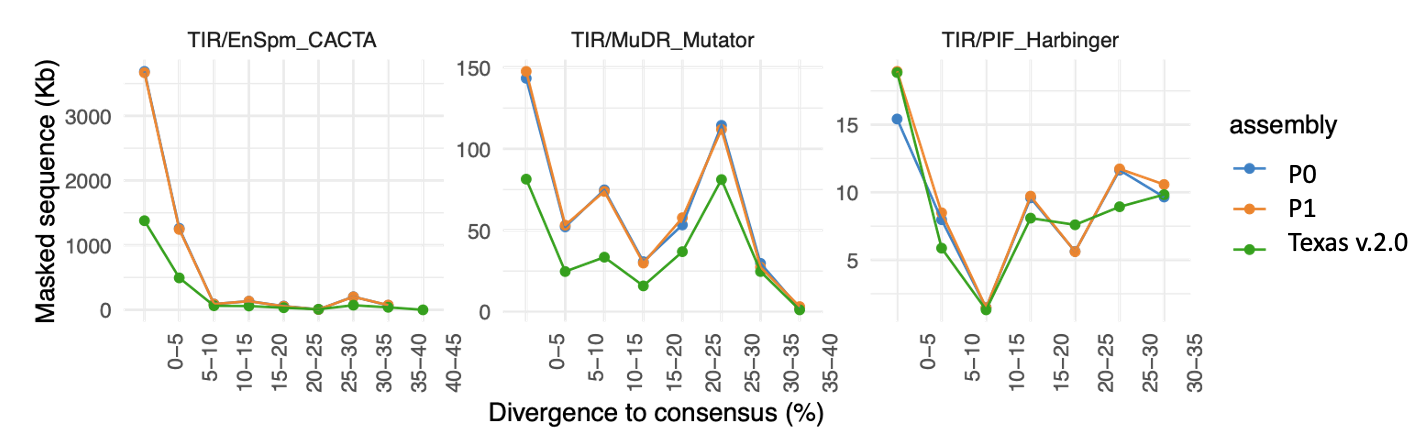


Supplementary Figure 8. Divergence of all TIR TE copies vs their respective consensus sequence.

Supplementary Figure 9. Length distribution of heterozygous insertions and deletions

TSD=CTAGT

P1_Chr05_657,234-658,033

P0_Chr05_620,728-631,827

gag

AP

RT

INT

98.7 %

TE_84251

P0-svim_asm.DEL.2261

96.0 %

97.0 %

TSD=GAGCT

P1_Chr05_2,997,899-2,998,706

P0_Chr05_2,977,997-2,989,809

RH

98.6%

TE_85837

P0-svim_asm.DEL.2273

97.5 %

98.5 %

TSD=GATGT

P1_Chr05_3,011,220-3,012,018

P0_Chr05_3,005,052-3,020,210

AP

RT

RH

INT

98.1 %

TE_85853

P0-svim_asm.DEL.2276

99.5 %

99.8 %

TSD=ACATA

P1_Chr05_3,908,154-3,908,934

P0_Chr05_3,986,805-3,997,890

gag

RT

RH

INT

99.4 %

TE_86503

P0-svim_asm.DEL.2293

98.3 %

97.5 %

TSD=AAGGG

P1_Chr05_4,021,026-4,021,838

P0_Chr05_4,112,044-4,123,264

98.5 %

TE_86566

P0-svim_asm.DEL.2295

99.3 %

98.2 %

TSD=TCCAA

P1_Chr05_4,545,655-4,546,449

P0_Chr05_4,782,411-4,791,932

IN

RH

RT

AP

gag

98.2 %

TE_87068

P0-svim_asm.DEL.2306

98.7 %

97,5 %

TSD=GATAC

P1_Chr05_10,121,731-10,122,516

P0_Chr05_10,377,551-10,384,924

gag

RT

RH

INT

99.5 %

TE_90424

P0-svim_asm.DEL.2340

99.0 %

99,8 %

TSD=GGTAA

P1_Chr05_10,185,266-10,186,069

P0_Chr05_10,447,818-10,454,137

gag

RT

RH

99.2 %

TE_90454

P0-svim_asm.DEL.2341

97.3 %

96,8 %

TSD=GAGAA

P1_Chr05_13,426,196-13,426,996

P0_Chr05_13,712,783-13,719,963

gag

RT

RH

INT

99.3 %

TE_93025

P0_svim_asm.DEL.2395

98.8 %

99.5 %

TSD=TGACT

P1_Chr05_15,139,742-15,140,541

P0_Chr05_15,460,431-15,467,728

gag

RT

RH

INT

99.3 %

TE_93634

P0-svim_asm.DEL.2414

98.5 %

99.8 %

TSD=TTTTC

P1_Chr05_17,174,723-17,175,542

P0_Chr05_17,561,609-17,567,337

gag

RT

RH

97.5 %

TE_94341

P0-svim_asm.DEL.2451

99.5 %

98.8 %

TSD=CTTTC

P1_Chr05_17,291,777-17,292,576

P0_Chr05_17,622,592-17,628,281

gag

RT

RH

100 %

TE_94374

P0-svim_asm.DEL.2455

100 %

100 %

TSD=CTCGT

P1_Chr05_18,462,419-18,463,228

P0_Chr05_18,772,122-18,778,492

gag

RT

RH

99.7 %

TE_94639

P0-svim_asm.DEL.2492

99.0 %

98.8 %

TSD=ATAGT

P1_Chr05_19,100,700-19,101,491

P0_Chr05_19,416,044-19,421,778

gag

RT

RH

98.9 %

TE_94853

P0-svim_asm.DEL.2508

97.3 %

97.5 %

TSD=AGTGC

P1_Chr05_20,116,799-20,117,592

P0_Chr05_20,429,600-20,438,221

gag

AP

99.8 %

TE_95323

P0-svim_asm.DEL.2521

99.5 %

100 %

TSD=GTTAC

P1_Chr05_20,347,234-20,348,029

P0_Chr05_20,642,567-20,657,957

RH

99.1 %

TE_95366

P0-svim_asm.DEL.2532

99.0 %

99.3 %

TSD=ACAAC

P1_Chr05_20,437,163-20,437,962

P0_Chr05_20,746,840-20,753,062

gag

RT

RH

97.8 %

TE_95398

P0-svim_asm.DEL.2535

100 %

100 %

TSD=GGAGA

P1_Chr05_20,604,012-20,604,813

P0_Chr05_20,914,974-20,920,826

gag

RT

RH

99.7 %

TE_95438

P0-svim_asm.DEL.2540

98.0 %

99.0 %

Supplementary Figure 10. Structure of insertions coinciding with polymorphic LTR-RT insertions. The identification number of the SV and the TE for each insertion is shown together with the positions in the P0 and P1 Texas v.3.0 assembly phases. A scheme of the LTR-RT and the position and sequence of the TSD (orange box) are shown. The sequence identity between the two LTRs of the LTR-RT and between the P0 and P1 sequences flanking the insertion (400nt) are also shown.

Supplementary Figure 11. Distribution of 3,148 TEs missing in Texas v.2.0 assembly but with conserved flanking regions based on the genome alignment.

Supplementary Figure S12. A) Distribution of heterozygous and homozygous LTR-retrotransposon insertion age. B) Population frequencies of heterozygous and homozygous TE insertions.


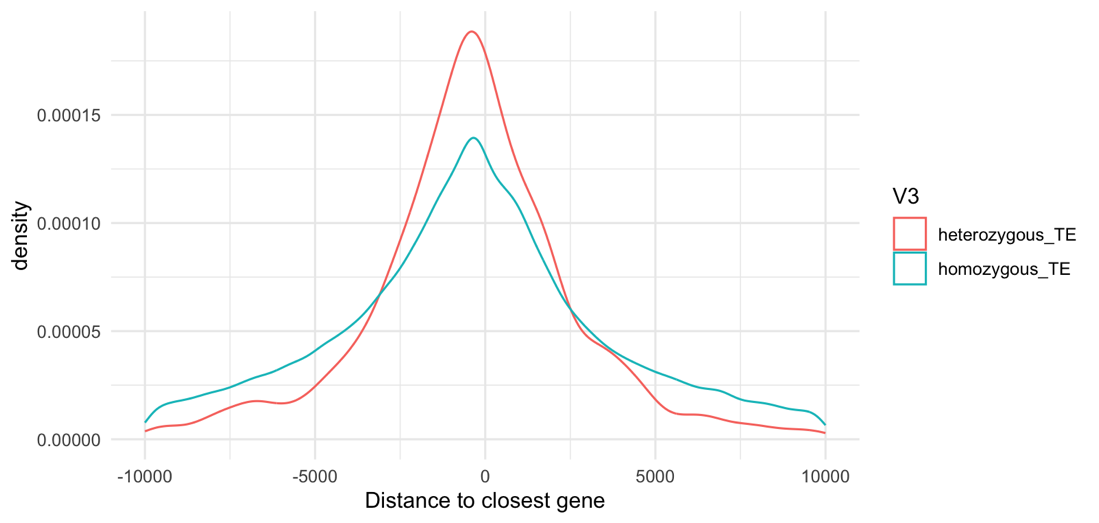


Supplementary figure 13. Distance of heterozygous and homozygous TE insertions to their closest gene. Elements overlapping gene regions are excluded.

Supplementary Table 1. Structural variants detected by SVIM-asm pipeline.

Supplementary Table 1. Structural variants detected by SVIM-asm

|  | P1-Texas v2.0 | P0 - Texas v2.0 | P1-P0 |
| --- | --- | --- | --- |
| Insertions | 5510 | 5543 | 3814 |
| Deletions | 5754 | 5725 | 3947 |
| Inversions | 9 | 10 | 5 |
| Tandem duplication | 112 | 109 | 100 |
| Interspersed duplication | 11 | 7 | 14 |
| Breakend candidates | 514 | 531 | 207 |

Supplementary Table 2. RNA-Seq datasets used for genome annotation.

| **Accession number** | **Species** | **Tissue** | **Source** | **Data type** |
| --- | --- | --- | --- | --- |
| LEAF_TEX1 | *P. dulcis* | leaf | *this study* | RNAseq |
| FLO_TEX1 | *P. dulcis* | flower | *this study* | RNAseq |
| FRU_TEX1 | *P. dulcis* | fruit | *this study* | RNAseq |
| SRR11251343 | *P. dulcis* | bud | NCBI SRA | RNAseq |
| SRR11251344 | *P. dulcis* | bud | NCBI SRA | RNAseq |
| SRR11251345 | *P. dulcis* | bud | NCBI SRA | RNAseq |
| SRR10189207 | *P. dulcis* | fruit | NCBI SRA | RNAseq |
| SRR10189208 | *P. dulcis* | fruit | NCBI SRA | RNAseq |
| SRR10189209 | *P. dulcis* | fruit | NCBI SRA | RNAseq |
| SRR6815287 | *P. dulcis* | root | NCBI SRA | RNAseq |
| SRR6815288 | *P. dulcis* | root | NCBI SRA | RNAseq |
| SRR6815289 | *P. dulcis* | root | NCBI SRA | RNAseq |
| RefSeq GCF_902201215.1 | *P. dulcis* | NA | NCBI genes | Protein |
| RefSeq GCF_000346465.2 | *P. persica* | NA | NCBI genes | Protein |
|  |  |  |  |  |

Supplementary Table 3. Heterozygous TE insertions

|  | **Detected** | | |
| --- | --- | --- | --- |
| **TE order** | **P0-specific** | **P1-specific** |  |
| TIR | 188 | 166 |  |
| MITE | 40 | 38 |  |
| RC/Helitron | 166 | 186 |  |
| LINE | 48 | 39 |  |
| LTR/Copia | 375 | 353 |  |
| LTR/Gypsy | 239 | 225 |  |
| LTR/unknown | 258 | 251 |  |

Supplementary Table 4. List of varieties used for TIP population analyses

| **SRA accession** | **Variety** | **Country** | **Continent** |
| --- | --- | --- | --- |
| ERR3366583 | Ai-2 | France | Europe |
| ERR3366585 | Belle dAurons-2 | France | Europe |
| ERR3366586 | Cristomorto | Italy | Europe |
| ERR3366588 | Desmayo largueta-2 | Spain | Europe |
| ERR3366589 | Falsa Barese | Italy | Europe |
| ERR3366590 | Genco | Italy | Europe |
| ERR3366591 | Marcona | Spain | Europe |
| ERR3366592 | Non-pareil | USA | North America |
| ERR3366593 | Ripon | USA | North America |
| ERR3366594 | Vivot | Spain | Europe |
| ERR4093803 | Texas | USA | North America |
| ERR4762264 | Del Cid | Spain | Europe |
| SRR3141032 | Shuang Guo | China | Asia |
| SRR3141040 | Zhi pi | China | Asia |
| SRR3141049 | Gong Ba Dan | China | Asia |
| SRR3141057 | Wan Feng | China | Asia |
| SRR3141065 | Ai Feng | China | Asia |
| SRR3141073 | Ba Dan Wang | China | Asia |
| SRR3141083 | Huang Shuang | China | Asia |
| SRR3141098 | A Yue Hun Zi | China | Asia |
| SRR3141113 | Tao Ba Dan | China | Asia |
| SRR3141181 | Da Ba Dan | China | Asia |
| SRR3141192 | Ye Er Qiang | China | Asia |
| SRR3141204 | Bian Zui He | China | Asia |
| SRR3141229 | Ao 2 # | USA | North America |
| SRR4036105 | #53 | USA | North America |
| SRR4036108 | Tardy Nonpareil | USA | North America |
| SRR4045222 | DPRU 1207.2 | USA | North America |
| SRR4045223 | Languedoc | USA | North America |
| SRR4045224 | DPRU 2331.9 | USA | North America |
| SRR4045225 | BE-1609 | USA | North America |
| SRR4045226 | Tuono | USA | North America |
| SRR4045227 | DPRU 2374.12 | USA | North America |
| SRR4045228 | Badam | USA | North America |
| SRR4045229 | DPRU 1462.2 | USA | North America |
| SRR7010336 | Lauranne | France | Europe |
| SRR7010337 | Alnem1 | France | Europe |
| SRR765679 | Ramillete | Spain | Europe |
| SRR765850 | D05-187 | Spain | Europe |
| SRR765861 | S3067 | Spain | Europe |
